# Protocol of a cluster randomized trial of an educational intervention to increase knowledge of living donor kidney transplant among potential transplant candidates

**DOI:** 10.1186/1471-2369-14-256

**Published:** 2013-11-19

**Authors:** Francis L Weng, Diane R Brown, John D Peipert, Bart Holland, Amy D Waterman

**Affiliations:** 1 Renal and Pancreas Transplant Division, Saint Barnabas Medical Center, 94 Old Short Hills Road, East Wing, Suite 305, Livingston, NJ 07039, USA; 2 Rutgers School of Public Health, 683 Hoes Lane West, Piscataway, NJ 08854, USA; 3 Department of Medicine, Division of General Medical Sciences, Washington University School of Medicine, Mailstop: 90-31-661, 660 S. Euclid Ave., St., Campus Box 8005, Louis, MO 63110-1093, USA; 4 New Jersey Medical School, Department of Preventive Medicine and Community Health, Rutgers University, Newark, NJ 07103, USA

**Keywords:** Kidney transplantation, Live kidney donor, Education, Clinical trial, Randomized trial, Cluster randomization

## Abstract

**Background:**

The best treatment option for end-stage renal disease is usually a transplant, preferably a live donor kidney transplant (LDKT). The most effective ways to educate kidney transplant candidates about the risks, benefits, and process of LDKT remain unknown.

**Methods/design:**

We report the protocol of the Enhancing Living Donor Kidney Transplant Education (ELITE) Study, a cluster randomized trial of an educational intervention to be implemented during initial transplant evaluation at a large, suburban U.S. transplant center. Five hundred potential transplant candidates are cluster randomized (by date of visit) to receive either: (1) standard-of-care (“usual”) transplant education, or (2) intensive education that is based upon the *Explore Transplant* series of educational materials. Intensive transplant education includes viewing an educational video about LDKT, receiving print education, and meeting with a transplant educator. The primary outcome consists of knowledge of the benefits, risks, and process of LDKT, assessed one week after the transplant evaluation. As a secondary outcome, knowledge and understanding of LDKT are assessed 3 months after the evaluation. Additional secondary outcomes, assessed one week and 3 months after the evaluation, include readiness, self-efficacy, and decisional balance regarding transplant and LDKT, with differences assessed by race. Although the unit of randomization is the date of the transplant evaluation visit, the unit of analysis will be the individual potential transplant candidate.

**Discussion:**

The ELITE Study will help to determine how education in a transplant center can best be designed to help Black and non-Black patients learn about the option of LDKT.

**Trial registration:**

Clinicaltrials.gov number NCT01261910

## Background

For patients with end-stage renal disease (ESRD), a kidney transplant from a living donor is usually the best treatment option. Living donor kidney transplants (LDKTs) and deceased donor kidney transplants (DDKTs) both improve ESRD patients’ survival and quality of life [1-4]. LDKT, however, offers several potential advantages compared to DDKT. LDKT allows transplant candidates to bypass the long DDKT waitlist, minimizes (and enables avoidance of) chronic dialysis and its associated morbidities [5], can be scheduled for a time convenient for the transplant recipient and living donor, and facilitates transplant among sensitized transplant candidates (who are often unable to otherwise receive a transplant) [6,7]. Furthermore, LDKT offers substantially better patient and allograft outcomes than DDKT [8]. For many patients, LDKT provides the only alternative to death on dialysis, while awaiting a DDKT [9,10].

Unfortunately, despite the many advantages of LDKT, Blacks with ESRD are less likely to receive LDKTs, compared to non-Blacks [11-13]. In 2011, Blacks made up 33.2% of the waiting list for DDKTs and received 32.2% of all DDKTs but received only 14.0% of all LDKTs [14]. This striking racial disparity stems from multiple factors and barriers [15,16]. One such barrier may be a need for more education and knowledge of LDKT and living donation, given the desire by Blacks (and non-Blacks) for such education and knowledge [17-19].

In addition to its advantages, LDKT has short-term and possibly long-term risks for living donors [20]. The short-term, peri-operative risks of donor nephrectomy (e.g. bleeding, infections, pneumothorax, and venous thromboembolism) resemble the risks of similar operations. Peri-operative death is very rare, occurring in approximately 0.03% of nephrectomies [21-23]. Long-term, living kidney donors may have increased risks of hypertension [24], proteinuria [25], and chronic kidney disease (CKD) [26,27]. These risks may be increased among donors who are Black, versus non-Black [23,26,27]. Despite these possible risks, living kidney donors appear to have the same rates of death and ESRD as the general population [10,23,28-30].

Given the substantial benefits of LDKT for the recipient and the possible long-term risks of nephrectomy for the living donor, kidney transplant candidates (and their living donors) should make informed choices regarding whether to pursue LDKT. Better education may increase CKD patients’ knowledge of LDKT. Among CKD patients, increased knowledge of LDKT has been associated with increased comfort in discussing LDKT with others and decreased concerns about live kidney donation [17].

The best source for accurate information about the process, risks, and benefits of LDKT is likely the local transplant center [31]. In the U.S., the Centers for Medicare and Medicaid Services has currently approved approximately 235 centers to perform kidney transplants [32]. Approved transplant centers must educate and inform patients about transplantation [33], but these centers vary greatly in how they deliver this education [34]. Each center educates patients using its own combination of print materials, videos, and group or one-on-one discussions with transplant personnel [34]. The best ways to provide education about LDKT remain unknown, so studies of potentially effective and replicable approaches to LDKT education are needed.

Therefore, we describe the protocol for the Enhancing Living Donor Kidney Transplant Education (ELITE) study, a cluster randomized clinical trial designed to test the impact of an educational intervention upon potential transplant candidates’ knowledge of LDKT. As mandated by the funding source, the federal Health Resources and Services Administration (HRSA), and grant program, the primary outcome of this study is knowledge of the benefits, risks, and process of live donor kidney transplant, rather than rates of actual LDKT [35].

## Methods/design

### Study design summary

**Figure 1:**
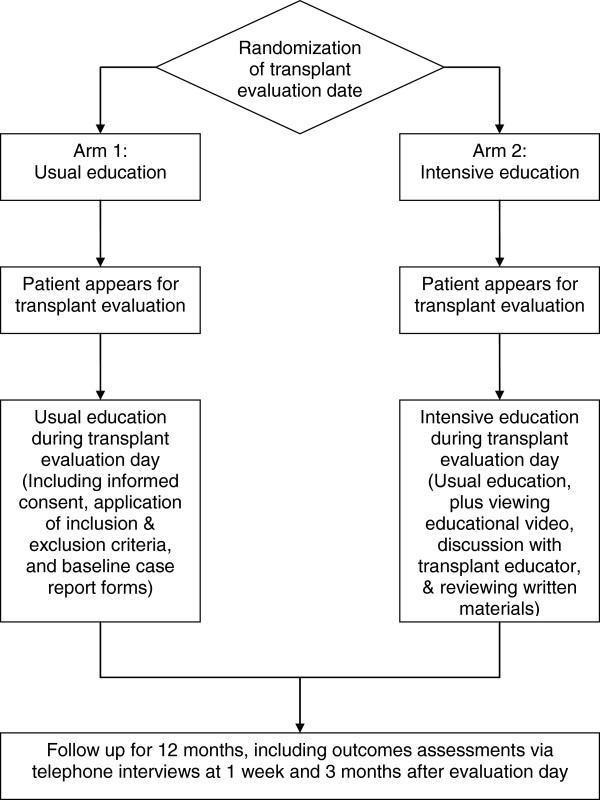
Flow diagram of study activities.

The ELITE study is a single-center, cluster randomized [36,37], parallel group clinical trial designed to test the effectiveness of an educational intervention [38] upon transplant candidates’ knowledge of LDKT (see Figure 1 for flow diagram and Additional file 1 for CONSORT 2010 checklist of information to include when reporting a cluster randomised trial). All study procedures were approved by the Institutional Review Boards at both Saint Barnabas Medical Center (SBMC, protocol number 2009–53) and the University of Medicine and Dentistry of New Jersey (protocol number 0120100096).

### Target population, setting, and inclusion/exclusion criteria

The targeted patient population consists of Black and non-Black persons with CKD who underwent evaluation for kidney transplant (potential transplant candidates) at Saint Barnabas Medical Center, a suburban transplant center and academic teaching hospital in Livingston, New Jersey, in the northeastern United States. We use the term “potential transplant candidates” to describe CKD patients who are referred for transplant and appear for evaluation by the transplant center. “Transplant candidates”, also known as “listed” or “actual” transplant candidates, are potential transplant candidates who are deemed suitable for transplant, made active on the waiting list for a DDKT, and able to receive a LDKT if they have a living donor. During the two years prior to the ELITE study, Saint Barnabas Medical Center evaluated approximately 700 potential transplant candidates per year.

Persons are eligible for the ELITE study if they meet the following inclusion criteria: (1) appear for initial kidney transplant evaluation at Saint Barnabas Medical Center; (2) are 18 years of age or older; (3) are able to provide informed consent; and (4) are able to speak, hear, and understand English. We exclude persons from the ELITE study if they: (1) have significant neurocognitive disability that would prevent participants from understanding the study or completing the questionnaires; (2) are unable to speak, hear, and understand English; (3) are visually impaired and unable to complete self-administered case report form; and (4) have a self-described unwillingness or inability to complete phone interviews.

### Study aims

Our primary aim is to determine the effects of an educational intervention (intensive transplant education), versus standard-of-care (usual) transplant education, upon transplant candidates’ knowledge of the benefits, risks, and process of LDKT, assessed one week after transplant evaluation.

As secondary aims, this study will also determine differences in readiness, self-efficacy, and decisional balance regarding transplant at one week after transplant evaluation, between patients who receive intensive, versus usual, transplant education. The study will also examine how knowledge, readiness, self-efficacy, and decisional balance change between one week and three months, in patients who receive intensive, versus usual, transplant education. Finally, the study will also determine how known barriers to LDKT, including Black race, less prior transplant education, and lower health literacy, act alone and in combination with the educational interventions to affect patients’ level of knowledge at one week and three months.

### Randomization

#### ***Randomization procedures and rationale for cluster randomization***

All the potential transplant candidates on a given evaluation day are cluster randomized to either standard-of-care transplant education (“usual” care or control, Arm 1) or intensive transplant education (experimental, Arm 2). At Saint Barnabas Medical Center, transplant education generally occurs thrice weekly, on Tuesdays, Wednesdays, and Fridays. Immediately before each week’s evaluations begin, we randomize each week’s evaluation days, using a computer-generated random number. Therefore, the unit of randomization will be the date of the transplant evaluation. As with most educational interventions, we are unable to blind or mask study personnel and participants regarding treatment allocation groups (usual care vs. intensive education). The study arm is unknown, however, at the time the potential transplant candidates scheduled their evaluations.

We chose cluster randomization to minimize contamination between study arms [39] as well as for practical administrative reasons. If we randomize at the level of the individual patient (potential transplant candidate), then on a given evaluation date, some potential transplant candidates will receive usual care (standard-of-care education), while others will receive intensive education (the intervention). Because potential candidates often talk and share information with each other throughout the evaluation day, randomization at the level of the individual patient will likely lead to significant contamination between study arms. Cluster randomization minimizes such contamination. In addition, by assigning all patients on a given date to receive the same education (usual or intensive), cluster randomization facilitates coordination of the transplant evaluation with performance of this research trial. Although the unit of randomization is the date of the transplant evaluation, the unit of analysis will be the individual potential transplant candidate.

### Standard-of-care (usual) transplant education (Arm 1)

During their transplant evaluation day, potential transplant candidates randomized to standard-of-care (usual) initial education will receive the standard transplant education and evaluation provided by SBMC. Family and friends are encouraged to accompany the patient to the evaluation. First, patients and their guests listen to and view a 90-minute slide presentation given by one of our trained transplant nurse coordinators. The presentation reviews:

• treatment options for chronic kidney disease, including LDKT and DDKT;

• the kidney transplant evaluation process;

• how the deceased donor waiting list works;

• the types of LDKTs and DDKTs. These slides succinctly review the benefits of LDKT, the workup of potential living donors, the types of living kidney donors, and alternative programs for LDKT;

• potential medical and psychosocial benefits and risks of kidney transplantation

• what to expect with the surgical procedure; and

• patients’ rights and responsibilities.

These topics must be included in the educational information provided to potential transplant candidates, as mandated by the Centers for Medicare and Medicaid Services [33].

After the presentation, potential transplant candidates are evaluated in private offices by members of the transplant team, including the transplant nurse coordinator, social worker, nephrologist, dietician, and financial coordinator. Potential transplant candidates meet separately with each transplant team member in a varying, unsystematic order, based on the availability of the team members. The research nurse or assistant also meets privately with each potential transplant candidate. Because the order in which potential transplant candidates meet with team members is unsystematic, the meeting between the research staff and the patient may occur at any point during the evaluation. The research staff member determines the patient’s eligibility for the study, obtains informed consent, and administers the initial questionnaires. The research nurse or assistant also sets an appointment date and time, at approximately one week after the transplant evaluation, for phone administration of follow-up questionnaires measuring knowledge and other outcomes.

### Intensive transplant education (experimental, Arm 2)

Potential transplant candidates who are cluster-randomized to intensive initial education (Arm 2) undergo the same education and evaluations as candidates in the usual education group. Additionally, study participants in the intensive education arm also undergo an intervention designed to increase knowledge of LDKT. This intervention is directed by the research nurse or assistant, who functions as a Transplant Educator. The intervention is based upon materials from the *Explore Transplant* series of kidney transplant education materials. When implemented in dialysis units, *Explore Transplant* has been shown to increase dialysis patients’ pursuit of DDKT and LDKT [40]. However, *Explore Transplant* has yet to be tested at a transplant center.

Our educational intervention consists of two parts, both implemented on the day of the transplant evaluation. First, study participants (together with any friends and family who accompanied them) view a 25-minute video, entitled “The experiences of living kidney donors”, from the *Explore Transplant* series of kidney transplant education materials [41,42]. This video, intended for potential transplant candidates, discusses how living donors came to their decision to donate; donors’ motivations and what they learned; facts about the donor evaluation, surgery, and recovery; risks to the living donors; and life after donation. The learning objectives of the video are to enable the viewer to understand the evaluation, surgery, and recovery process of living donors; learn possible risks that living donors face and how often these risks occur; learn the advantage of living donor kidneys versus deceased donor kidneys; and learn ways family member and friends can provide support to transplant candidates. The video features the stories of actual living kidney donors and presents accurate medical facts about LDKT, presented by medical professionals.

Second, after viewing the video, the study participants meet privately with the trained Transplant Educator for a fifteen-minute meeting to:

• Answer any questions that patients may have had, based upon what they saw in the video;

• Explore with patients their reasons for wanting a kidney transplant;

• Explore with patients their current situation with CKD/ESRD;

• Examine how the patients’ CKD/ESRD affects their family and friends;

• Answer any questions or concerns about transplant that the patient has;

• Review a brochure from *Explore Transplant*, “Why Kidney Patients Get Transplants/Why People Donate Their Kidneys”;

• Review a fact sheet from *Explore Transplant*, “Possible Risks to Living Donors”; and

• Review a fact sheet from *Explore Transplant*, “Living Donors’ Evaluation, Surgery, and Recovery”.

Because the order in which potential transplant candidates meet with team members is unsystematic, the viewing of the video and meeting with the Transplant Educator occur at any point during the transplant evaluation.

### Outcomes

#### ***Measurement timepoints***

After the transplant evaluation, we measure knowledge, readiness, self-efficacy, and decisional balance regarding transplant (DDKT and LDKT) (Table 1). To measure these characteristics, we administer telephone questionnaires to study participants at 1 week and 3 months after the transplant evaluation. These questionnaires are administered by research personnel who are not involved in participant recruitment and who are blinded to the study arm.

**Table 1:** Data collection

	**Day of evaluation**	**1 week later**	**3 months later**	**12 months later**
**Transplant information**				
Demographic and medical characteristics	X			
Prior discussions with physicians regarding transplant	X			
Prior education regarding transplant	X			
Willingness to accept LDKT		X	X	
Actions to find living donors		X	X	
Placement on DDKT waiting list for transplant (and reasons for non-listing)				X
Mortality status				X
**Health literacy**				
Newest Vital Sign	X			
Single-item questions regarding use of surrogate reader and confidence with filling out forms	X			
**Outcomes**				
Knowledge of transplant		X	X	
Readiness to pursue transplant		X	X	
Self-efficacy regarding transplant		X	X	
Decisional balance regarding transplant		X	X	

We are unable to measure study participants’ “baseline” knowledge, readiness, self-efficacy, and decisional balance regarding transplant, prior to starting the evaluation. This inability stems from human subjects IRB restrictions that prevent us from administering questionnaires by telephone to persons who have not provided written informed consent. In addition, on the day of the actual transplant evaluation, obtaining individual written informed consent from up to six transplant candidates, prior to the delivery of any actual education, is also infeasible. Therefore, due to human subjects limitations and practical concerns, we are unable to obtain baseline measures (of knowledge, readiness, self-efficacy, and decisional balance regarding transpland and LDKT) prior to the start of usual or intensive transplant education.

#### ***Primary outcome: knowledge of LDKT at 1 week after the transplant evaluation***

The primary study outcome is knowledge of the benefits, risks, and process of LDKT, one week after the transplant evaluation. Knowledge is measured using a 20-item questionnaire that contains 12 true-false and 8 multiple-choice questions. This questionnaire is being used in other studies of LDKT and live kidney donation [42].

#### ***Secondary outcome: change in knowledge of LDKT at 3 months after the transplant evaluation***

As a secondary outcome, we also assess change in knowledge of LDKT three months after the transplant evaluation. Knowledge at three months is assessed using the same 20-item questionnaire that we use at 1 week after the transplant evaluation. LDKT knowledge “difference” scores will be calculated by subtracting the 1 week post-baseline LDKT knowledge scores from the 3 month post-baseline scores.

#### ***Other secondary outcomes***

The other secondary outcomes include: (1) readiness to pursue transplant and LDKT; (2) self-efficacy regarding transplant and LDKT; and (3) decisional balance regarding transplant and LDKT. These outcomes are also assessed by phone-administered questionnaires, at one week and three months post-evaluation. We will calculate self-efficacy and decisional balance “difference” scores by subtracting the 1 week post-baseline LDKT knowledge scores from the 3 month post-baseline scores.

#### ***Readiness to pursue DDKT and LDKT***

Readiness to pursue DDKT and LDKT are assessed using the Stages of Change, from Prochaska’s Transtheoretical Model [43,44]. For readiness to pursue DDKT, study participants choose whether there are in the Pre-Contemplation, Contemplation, Preparation, Action, or Maintenance stages (see Table 2 for descriptions of stages). Similarly, for readiness to pursue LDKT, study participants self-identify whether they are in Pre-Contemplation, Contemplation, Preparation, or Action (Table 2).

**Table 2:** Statements that describe readiness (Stage of Change) for deceased donor and living donor kidney transplant

**Stage of Change**	**Readiness for deceased donor kidney transplant**	**Readiness for live donor kidney transplant**
**Pre-contemplation**	“I am NOT considering in the next 6 months getting a deceased donor transplant”	“I am NOT considering in the next 6 months getting a living donor transplant”
**Contemplation**	“I am considering taking actions in the next 6 months to get a deceased donor transplant”	“I am considering taking actions in the next 6 months to get a living donor transplant”
**Preparation**	“I am preparing to take actions in the next 30 days to get a deceased donor transplant”	“I am preparing to take actions in the next 30 days to get a living donor transplant”
**Action**	“I am undergoing transplant evaluation to get a deceased donor transplant”	“I am taking actions to get a living donor transplant”
**Maintenance**	“I am listed and waiting to get a deceased donor transplant”	None

To further assess readiness to pursue LDKT, we also assess Stage of Change regarding (1) asking a family or friend to consider living donation and (2) accepting an offer of a live donor kidney (among the subset of study participants who had received an offer of a live donor kidney).

#### ***Self-efficacy (transplant confidence)***

We measure participants’ self-efficacy (transplant confidence), which reflects their confidence that they can enact and sustain a behavior change [45] (in this case, the behavior change is receipt of a transplant or LDKT). We assess study participants’ confidence in their ability to pursue transplant and LDKT in difficult situations. For transplant in general, we ask “How confident are you that you could get a transplant even if…” for eight items and scenarios, such as “…your friends and family were unsupportive of you getting a transplant”. Similarly, six self-efficacy questions focus upon LDKT and ask “How confident are you that you could get a living donor transplant even if…”, followed by items and scenarios such as “…you don’t know anyone who might be a living donor for you”. Responses are on a 5-point Likert scale from “Not at all confident” to “Completely confident”.

#### ***Decisional balance (Pros and Cons of DDKT and LDKT)***

Decisional balance measures how patients are weighing the advantages and disadvantages (pros and cons) of transplant. We ask patients to rate the importance of possible positive and negative items related to patients’ decisions about DDKT and LDKT. We include 12 items regarding transplant in general and 12 items regarding LDKT. For each item, we ask, “How important is this statement to your decision about transplant/living donor transplant?” Sample statements include “I would live a longer life with a transplant” (Pro of transplant) and “I will feel guilty having someone donate to me” (Con of LDKT). Responses are rated on a 5-point Likert scale from “Not important” to “Extremely important”.

### Other collected variables

#### ***Prior actions taken to learn about transplant***

On the day of the transplant evaluation, we ask study participants about prior actions they had taken to learn about kidney transplant. These prior actions include: reading brochures about kidney transplant, reading brochures about LDKT, browsing Internet websites about kidney transplant, watching television or videos about kidney transplant, attending kidney disease support groups, talking to recipients of a kidney transplant, talking to doctors and medical staff about transplant, and talking to family and friends about transplant.

#### ***Health literacy***

On the day of the transplant evaluation, we assess study participants’ health literacy in two ways. First, we administer the Newest Vital Sign (NVS), a validated in-person measure of health literacy and numeracy [46]. The NVS assesses functional health literacy and numeracy and is scored from zero to six, with higher scores reflecting better health literacy. Scores of zero to three suggest limited health literacy [46].

Second, we use screening questions to inquire about the participants’ ability to read, comprehend, and fill out hospital forms [47,48]. These screening questions ask the study participant (1) “How often do you have problems learning about your medical condition because of difficulty understanding written information?” (always, often, sometimes, occasionally, or never); (2) “How often do you have someone help you read hospital materials?” (always, often, sometimes, occasionally, or never); and (3) “How confident are you filling out medical forms by yourself?” (extremely, quite a bit, somewhat, a little bit, or not at all). These questions are effective in identifying persons with limited health literacy [49].

### Final follow-up

After the 1-week and 3-month telephone interviews, study participants are followed for 12 months after their transplant evaluation. At that point, based upon our medical records, we determine whether the potential transplant candidate is placed on the waiting list at our center (and if not placed on the list, then the reason why) and whether the study participant is alive or not.

### Reimbursements and incentives for study participants

At the transplant evaluation, study participants receive a $15 gift card to a local supermarket, gas station, or retailer, as an incentive for study participation in either usual or intensive education. After the completion of the 3-month phone interview, a $10 gift card is mailed to the study participants. No additional incentives were given for completion of the 1-week or 3-month phone interviews.

### Statistical analysis

The initial data analysis will be descriptive. Participants’ baseline variables (demographics, medical characteristics, providers, etc.) will be described, using frequencies for categorical variables (such as race), and mean, median, range, standard deviation, and standard error of the means for continuous variables (such as age). Scatter plots of continuous variables and plots of means versus levels of categorical variables will be examined to assess bivariate relationships.

To determine whether randomization resulted in the desired equal distributions in the arms of the study, we will initially compare frequencies, means, and medians for baseline variables across the two study arms (usual vs. intensive education). Because the data are clustered by evaluation session, all significance tests (bivariate and multivariable statistical tests) will use generalized estimating equations (GEE), which account for variance determined by the cluster or “design effect” [50,51]. The analysis will use a per-protocol analysis. As a sensitivity analysis, we will perform an intent-to-treat analysis [52], in which we use multiple imputation for data that are missing. Multiple imputation allows intent-to-treat analysis by permitting inclusion in the analysis of all enrolled study participants [53].

#### ***Primary analysis***

To evaluate the effects of the intensive versus usual transplant education, upon LDKT knowledge one week after the transplant evaluation, we will compare overall knowledge scores between groups of candidates. These knowledge scores will be calculated by totaling the number of correct responses (range 0–20). We will compare knowledge scores between the usual vs. intensive education groups using the two-sample t-test with GEE. Randomization assignment (usual vs. intensive education) will be the independent variable. To adjust for any baseline characteristics that are unequally distributed between the two study arms, and to examine and control for the effects of confounders and other covariates, we will use multivariable linear regression models with GEE to determine differences in knowledge between the two study arms (usual vs. intensive education).

#### ***Secondary analyses***

As a secondary outcome, we will also assess change in transplant knowledge from 1 week post-evaluation to approximately three months after the transplant evaluation. In this analysis, the outcome will be measured as “difference scores” (the 1 week knowledge score subtracted from the three month knowledge scores) using linear regression with GEE and adjusting for unequally distributed baseline characteristics [54].

We will determine the impact of the study intervention upon the other secondary outcomes: (1) readiness to pursue transplant and LDKT at 1 week post-evaluation; (2) self-efficacy regarding transplant and LDKT at 1 week post-evaluation; (3) decisional balance regarding transplant and LDKT at 1 week post-evaluation; and (4) changes in readiness, self-efficacy, and decisional balance regarding transplant and LDKT, from 1 week to 3 months post-evaluation. Self-efficacy, decisional balance, and change in self-efficacy and decisional balance are measured on a continuous scale, so they will also be analyzed with linear regression with GEE. Readiness to pursue transplant and LDKT will be analyzed as dichotomous outcomes, where the probability of patients being in Action or Maintenance vs. the probability of being in Pre-contemplation, Contemplation, or Preparation will be modeled with logistic regression with GEE. The tests for all secondary outcomes will also be adjusted for any unbalanced baseline characteristics.

Finally, to examine how key barriers to LDKT work alone and in combination with our intervention to impact LDKT knowledge, we will test the independent effects of race, previous actions taken to learn about transplant, and health literacy on knowledge at 1 week post-baseline. We will also test whether these variables interact with the intervention to effect knowledge of LDKT. We will model knowledge of LDKT as the dependent variable using multivariable linear regression models with GEE. To fit the models, we will use forward selection procedures where independent variables that are univariately significant at P < 0.20 will be eligible for inclusion in the multivariable model. Those variables significant at P < 0.05 in the multivariable models will be retained [55].

### Power and sample size calculations

Our primary analysis will determine whether the study intervention is associated with increased knowledge of LDKT, one week after the transplant evaluation. Because the randomization of patients is clustered by evaluation date, sample size and power calculations for testing the effect of the intervention must be based on the number of evaluation days randomized to each condition, the number of patients per evaluation day, the expected intra-class correlation (ICC) among patients within a cluster (ICC indicates within-group similarity on the outcome), and the estimated difference between conditions in, and variability of, transplant knowledge scores. Based on previous research [40], we estimate that patients in the intervention arm will have 10-20% higher transplant knowledge at 1 week post-baseline than patients in the standard-of-care arm. Assuming a conservative ICC of 0.4, we calculated that with 250 patients in each of the two study arms from approximately 100 evaluation days per study arm (2–3 patients per evaluation day), a two-sample t-test with type 1 error of 0.05 would have at least 95% power to detect a 10-20% difference in knowledge scores. In summary, to have sufficient power to detect differences in the primary and secondary outcomes, this study should include 250 patients in each education condition at 1 week post-evaluation.

## Discussion

We designed the ELITE study to determine whether an educational intervention, implemented on the day of the transplant evaluation at the transplant center, increases knowledge of LDKT among potential transplant candidates, assessed one week and 3 months after the evaluation. Additional secondary outcomes include readiness, self-efficacy, and decisional balance regarding transplantation and LDKT, assessed one week and 3 months after the evaluation. We also will compare the effectiveness of the different educational interventions for Black and non-Black patients and patients of varying levels of health literacy.

This study is notable for testing an intervention that is physically delivered at the transplant center, in a method consistent with standard clinical care. If effective, more intensive transplant education could be replicated and easily incorporated into transplant evaluations in the approximately 235 transplant centers nationwide. Surprisingly, few interventions have targeted CKD patients being evaluated at the transplant center [42,56]. Instead, most prior interventions have targeted CKD patients in nephrologists’ offices [57,58], dialysis units [59,60], and patients’ homes [56,61]. By bringing together CKD patients who have some preexisting interest in transplant, the transplant evaluation, offers an opportunity to efficiently and effectively educate these patients about LDKT.

Our study design has several potential limitations. Our educational intervention is a relatively brief, one-time event that occurs solely at the transplant evaluation. Typically, the study participants in the intensive education arm will spend less than one hour watching videos and meeting with the Transplant Educator. An intervention that includes additional education sessions (e.g. after the transplant evaluation) may be more effective, albeit less practical. In addition, all study participants in the intervention arm receive the same intervention. If we were able to measure study participants’ knowledge and interest in LDKT prior to the transplant evaluation, then we could tailor the intervention for each participant’s level of readiness.

In conclusion, the results of the ELITE study have the potential to guide clinical care and patient education in kidney transplant centers. Studies of behavioral and educational interventions implemented during the transplant evaluation at the transplant center will enable the transplant community to determine the best ways to educate the CKD population regarding transplant.

## Abbreviations

LDKT: Live donor kidney transplant; DDKT: Deceased donor kidney transplant; ESRD: End-stage renal disease; CKD: Chronic kidney disease; NVS: Newest vitals sign; SBMC: Saint Barnabas Medical Center; ELITE study: Enhancing living donor kidney transplant education study.

## Competing interests

ADW developed the *Explore Transplant* educational materials and videos used in this study.

## Authors’ contributions

FLW conceived, designed, and conducted the study and drafted the manuscript. ADW contributed to the conception and design of the study and *Explore Transplant* educational materials and to the drafting of the manuscript. BH contributed to the conception and design of the study and to the drafting of the manuscript. JDP contributed to the design of the study and to the drafting of the manuscript. DRB contributed to the conception and design of the study and to the drafting of the manuscript. All authors approved the manuscript version.

## Pre-publication history

The pre-publication history for this paper can be accessed here:

http://www.biomedcentral.com/1471-2369/14/256/prepub

## Supplementary Material

Additional file 1 CONSORT 2010 checklist of information to include when reporting a cluster randomised trial. Click here for file
