# Supplementary material for: Protocol of a cluster randomized trial of an educational intervention to increase knowledge of living donor kidney transplant among potential transplant candidates
Source: BMC Nephrol. 2013 Nov 19;14:256. doi: 10.1186/1471-2369-14-256 (PMC3840671; doi:10.1186/1471-2369-14-256)
Supplement: Additional file 1 — CONSORT 2010 checklist of information to include when reporting a cluster randomised trial. [file 1471-2369-14-256-S1.doc]

**CONSORT 2010 checklist of information to include when reporting a cluster randomised trial**

| **Section/Topic** | **Item No** | **Standard Checklist item** | **Extension for cluster designs** | **Page No *** |
| --- | --- | --- | --- | --- |
| **Title and abstract** | | | |  |
|  | 1a | Identification as a randomised trial in the title | Identification as a cluster randomised trial in the title | **1** |
|  | 1b | Structured summary of trial design, methods, results, and conclusions (for specific guidance see CONSORT for abstracts) | See table 2 | **3** |
| **Introduction** | | | |  |
| **Background and objectives** | 2a | Scientific background and explanation of rationale | Rationale for using a cluster design | **5-7** |
|  | 2b | Specific objectives or hypotheses | Whether objectives pertain to the the cluster level, the individual participant level or both | **6-7** |
| **Methods** | | | |  |
| **Trial design** | 3a | Description of trial design (such as parallel, factorial) including allocation ratio | Definition of cluster and description of how the design features apply to the clusters | **6-9** |
|  | 3b | Important changes to methods after trial commencement (such as eligibility criteria), with reasons |  | **N/A** |
| **Participants** | 4a | Eligibility criteria for participants | Eligibility criteria for clusters | **7-8** |
|  | 4b | Settings and locations where the data were collected |  | **7-8** |
| **Interventions** | 5 | The interventions for each group with sufficient details to allow replication, including how and when they were actually administered | Whether interventions pertain to the cluster level, the individual participant level or both | **10-13** |
| **Outcomes** | 6a | Completely defined pre-specified primary and secondary outcome measures, including how and when they were assessed | Whether outcome measures pertain to the cluster level, the individual participant level or both | **13-16** |
|  | 6b | Any changes to trial outcomes after the trial commenced, with reasons |  | **N/A** |
| **Sample size** | 7a | How sample size was determined | Method of calculation, number of clusters(s) (and whether equal or unequal cluster sizes are assumed), cluster size, a coefficient of intracluster correlation (ICC or *k*), and an indication of its uncertainty | **21** |
|  | 7b | When applicable, explanation of any interim analyses and stopping guidelines |  | **N/A** |
| **Randomisation:** | | | |  |
| **Sequence generation** | 8a | Method used to generate the random allocation sequence |  | **9** |
|  | 8b | Type of randomisation; details of any restriction (such as blocking and block size) | Details of stratification or matching if used | **9-10** |
| **Allocation concealment mechanism** | 9 | Mechanism used to implement the random allocation sequence (such as sequentially numbered containers), describing any steps taken to conceal the sequence until interventions were assigned | Specification that allocation was based on clusters rather than individuals and whether allocation concealment (if any) was at the cluster level, the individual participant level or both | **9** |
| **Implementation** | 10 | Who generated the random allocation sequence, who enrolled participants, and who assigned participants to interventions | Replace by 10a, 10b and 10c | **--** |
|  | 10a |  | Who generated the random allocation sequence, who enrolled clusters, and who assigned clusters to interventions | **9-10** |
|  | 10b |  | Mechanism by which individual participants were included in clusters for the purposes of the trial (such as complete enumeration, random sampling) | **9-10** |
|  | 10c |  | From whom consent was sought (representatives of the cluster, or individual cluster members, or both), and whether consent was sought before or after randomisation | **11** |
|  |  |  |  |  |
| **Blinding** | 11a | If done, who was blinded after assignment to interventions (for example, participants, care providers, those assessing outcomes) and how |  | **9, 13-14** |
|  | 11b | If relevant, description of the similarity of interventions |  | **N/A** |
| **Statistical methods** | 12a | Statistical methods used to compare groups for primary and secondary outcomes | How clustering was taken into account | **18-21** |
|  | 12b | Methods for additional analyses, such as subgroup analyses and adjusted analyses |  | **18-21** |
| **Results** | | | |  |
| **Participant flow (a diagram is strongly recommended)** | 13a | For each group, the numbers of participants who were randomly assigned, received intended treatment, and were analysed for the primary outcome | For each group, the numbers of clusters that were randomly assigned, received intended treatment, and were analysed for the primary outcome | **N/A- this is a protocol paper without results** |
|  | 13b | For each group, losses and exclusions after randomisation, together with reasons | For each group, losses and exclusions for both clusters and individual cluster members | **N/A- this is a protocol paper** |
| **Recruitment** | 14a | Dates defining the periods of recruitment and follow-up |  | **N/A- this is a protocol paper** |
|  | 14b | Why the trial ended or was stopped |  | **N/A- this is a protocol paper** |
| **Baseline data** | 15 | A table showing baseline demographic and clinical characteristics for each group | Baseline characteristics for the individual and cluster levels as applicable for each group | **N/A- this is a protocol paper** |
| **Numbers analysed** | 16 | For each group, number of participants (denominator) included in each analysis and whether the analysis was by original assigned groups | For each group, number of clusters included in each analysis | **N/A- this is a protocol paper** |
| **Outcomes and estimation** | 17a | For each primary and secondary outcome, results for each group, and the estimated effect size and its precision (such as 95% confidence interval) | Results at the individual or cluster level as applicable and a coefficient of intracluster correlation (ICC or k) for each primary outcome | **N/A- this is a protocol paper** |
|  | 17b | For binary outcomes, presentation of both absolute and relative effect sizes is recommended |  | **N/A- this is a protocol paper** |
| **Ancillary analyses** | 18 | Results of any other analyses performed, including subgroup analyses and adjusted analyses, distinguishing pre-specified from exploratory |  | **N/A- this is a protocol paper** |
| **Harms** | 19 | All important harms or unintended effects in each group (for specific guidance see CONSORT for harms[[1]](#endnote-2)) |  | **N/A- this is a protocol paper** |
| **Discussion** | | | |  |
| **Limitations** | 20 | Trial limitations, addressing sources of potential bias, imprecision, and, if relevant, multiplicity of analyses |  | **N/A- this is a protocol paper** |
| **Generalisability** | 21 | Generalisability (external validity, applicability) of the trial findings | Generalisability to clusters and/or individual participants (as relevant) | **N/A- this is a protocol paper** |
| **Interpretation** | 22 | Interpretation consistent with results, balancing benefits and harms, and considering other relevant evidence |  | **N/A- this is a protocol paper** |
| **Other information** | | |  |  |
| **Registration** | 23 | Registration number and name of trial registry |  | **3** |
| **Protocol** | 24 | Where the full trial protocol can be accessed, if available |  | **N/A** |
| **Funding** | 25 | Sources of funding and other support (such as supply of drugs), role of funders |  | **6-7** |

** Note: page numbers optional depending on journal requirements*

1. [↑](#endnote-ref-2)
